# In crystallo observation of active site dynamics and transient metal ion binding within DNA polymerases

**DOI:** 10.1063/4.0000187

**Published:** 2023-06-15

**Authors:** Caleb Chang, Grace Zhou, Yang Gao

**Affiliations:** Department of Biosciences, Rice University, Houston, Texas 77005, USA

## Abstract

DNA polymerases are the enzymatic catalysts that synthesize DNA during DNA replication and repair. Kinetic studies and x-ray crystallography have uncovered the overall kinetic pathway and led to a two-metal-ion dependent catalytic mechanism. Diffusion-based time-resolved crystallography has permitted the visualization of the catalytic reaction at atomic resolution and made it possible to capture transient events and metal ion binding that have eluded static polymerase structures. This review discusses past static structures and recent time-resolved structures that emphasize the crucial importance of primer alignment and different metal ions binding during catalysis and substrate discrimination.

DNA polymerases are essential enzymatic catalysts that synthesize DNA. Based on their sequence homology, DNA polymerases are divided into A, B, C, D, X, Y, reverse transcriptase (RT), and primase and polymerase (PrimPol) families. As a diverse group of enzymes, they function during different stages of DNA replication and repair, prefer different DNA substrates, and vary in subunit compositions, catalytic rate, fidelity, and processivity (Wu *et al.*, 2017; Yang and Gao, 2018). Despite the diversity in their various biological roles and biochemical properties, early crystal structures revealed that DNA polymerases resemble a right-hand shape with the active site residing in the palm domain, nucleotide binding site at the finger domain, and the DNA binding site at the thumb domain [Fig. 1(a)] (Steitz, 1999). The finger domain in replicative DNA polymerases undergoes an open-to-close conformational change upon correct nucleotide binding, whereas some polymerases specialized in translesion DNA synthesis do not exhibit finger domain movement (Johnson, 1993; Steitz, 1999; Yang, 2014). DNA synthesis requires divalent metal ions as cofactors (Yang, 2014). Two-metal ions (Me^2+^) are coordinated by conserved negatively charged residues within the polymerase active site [Fig. 1(b)]. The A site metal ion (Me^2+^_A_) binds near the primer terminus of the DNA duplex, while the B site metal ion (Me^2+^_Β_) is coordinated by the incoming nucleotide phosphate oxygens. Much efforts in capturing static crystal structures of polymerases complexed with various DNA substrates, drug compounds, and damaged DNA lesions have allowed enzymologists to further understand the function of polymerases during DNA replication and repair (Wu *et al.*, 2017; Yang and Gao, 2018). The substantial plasticity of the active site when polymerases interact with different DNA templates, nucleotide substrates, and metal ions suggests the existence of rich dynamic events and their critical roles during DNA synthesis.

**FIG. 1.**
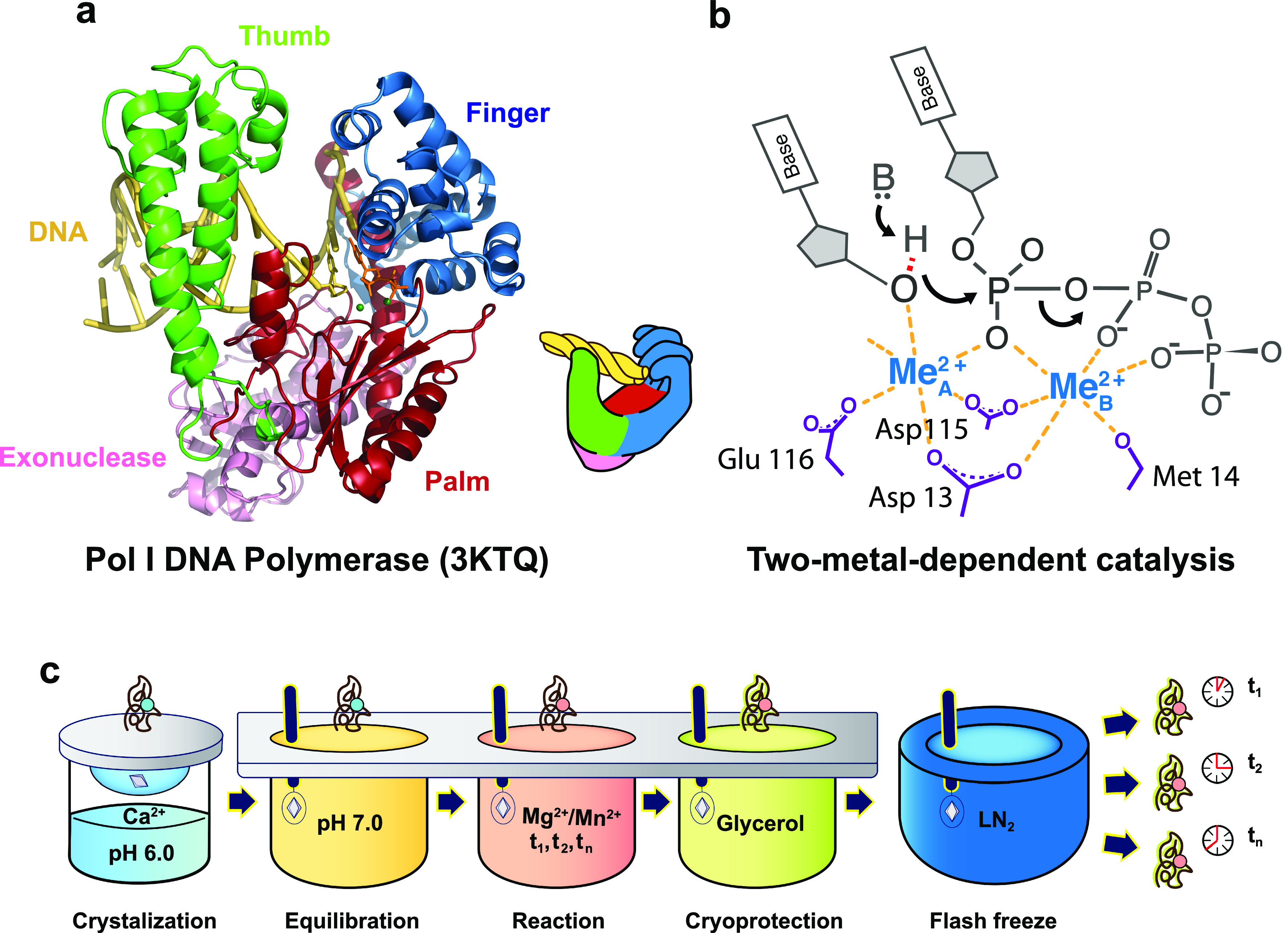
Unveiling the catalytic reaction of DNA polymerases by time-resolved crystallography. (a) Right-hand architecture of DNA polymerase I with the DNA colored in yellow and the substrate nucleotide colored in orange. The Me^2+^_A_ and Me^2+^_B_ are colored in green. The palm, finger, thumb, and exonuclease domains are colored in red, blue, green, and pink, respectively. (b) Schematic of the two-metal-dependent catalytic mechanism. The general base is denoted as B. The labeled residues are based on structures of Pol η. **c**, Metal ion soaking setup for *in crystallo* observation of DNA synthesis. The crystal of a ternary polymerase complex is grown with Ca^2+^ in the hanging drop. The polymerase crystal is looped and first soaked in the equilibration buffer (crystallization condition with a pH of 7.0) to change the pH to pH 7.0. Afterward, the *in crystallo* reaction is initiated by looping and soaking the crystal in the reaction buffer (equilibration buffer that includes Mg^2+^ or Mn^2+^). After soaking for a brief period of time, the crystal is looped and soaked in the cryoprotection buffer (reaction buffer that includes 20% glycerol) for 1 s. Then, the crystal is rapidly immersed in liquid nitrogen and stored for x-ray analysis.

Kinetic methods have elucidated the overall catalytic process of DNA synthesis and suggested that most polymerases catalyze DNA synthesis through similar kinetic pathways (Johnson, 1993; Joyce and Benkovic, 2004; Raper *et al.*, 2018; Wu *et al.*, 2017; Yang and Gao, 2018). First, DNA and the incoming nucleotide bind subsequently to the polymerase active site. Afterward, the 3′-OH of the primer becomes deprotonated and attacks the α-phosphate of the incoming nucleotide, followed by sequential release of the Me^2+^ and pyrophosphate. At last, the release or translocation of DNA prepares the polymerase for the next round of DNA synthesis. Pre-steady state kinetics investigating nucleotide incorporation and misincorporation revealed that an anomalous conformational step right before and after nucleophilic attack may be the rate-limiting step in DNA synthesis and can contribute to polymerase fidelity (Patel *et al.*, 1991; Wong *et al.*, 1991). Early studies attributed this rate-limiting step to the open-to-close conformational change of the finger domain; however, the finger movement has been found to be excessively fast to be considered rate-limiting (Dunlap and Tsai, 2002; Rothwell *et al.*, 2005; Tsai and Johnson, 2006). Some experiments further suggested that a “micro” closing conformational movement is rate-limiting, while others argue that no such conformational changes are needed to explain the observed kinetic data in substrate discrimination (Johnson, 2010; Oertell *et al.*, 2016; Raper *et al.*, 2018; Raper and Suo, 2016; Showalter and Tsai, 2002; Tsai and Johnson, 2006; Vande Berg *et al.*, 2001). Direct visualization of the polymerase catalysis at atomic resolution is, thus, critical to illustrate the dynamic process of DNA synthesis and to clarify the role of the conformational checkpoint for catalysis and incorrect substrate discrimination.

Diffusion-based time-resolved crystallography has made it possible to observe transient events that occur during DNA synthesis *in crystallo* at atomic resolution (Chim *et al.*, 2021; Freudenthal *et al.*, 2013; Gao and Yang, 2016; Gregory *et al.*, 2021; Nakamura *et al.*, 2012; Samara and Yang, 2018; Vyas *et al.*, 2015). Initial *in crystallo* studies observed a growing DNA chain after DNA translocation at high resolution by soaking A-family polymerase I Klenow fragment crystals in nucleotide-containing buffer (Johnson *et al.*, 2003). However, rapid events immediately before and after the nucleophilic attack were not captured due to the slow diffusion rate of the nucleotide. Recently, an *in crystallo* reaction method that involves soaking DNA-polymerase-nucleotide (ternary) complex crystals in Me^2+^-rich buffer was developed (Chang *et al.*, 2022; Gao and Yang, 2016; Gregory *et al.*, 2021; Nakamura *et al.*, 2012; Samara *et al.*, 2017; Yang *et al.*, 2017). The DNA synthesis reaction in polymerase ternary complex crystals with inhibitory metal ion Ca^2+^ is initiated by the diffusion and binding of Mg^2+^ and then halted by flash-quenching with liquid nitrogen freezing [Fig. 1(c)]. Due to the small size and the fast diffusion rate of metal ions, Mg^2+^-diffusion-based *in crystallo* studies on X-family Pols β, λ, and μ and Y-family Pol η have revealed transient mechanistic events immediately preceding the nucleophilic attack as well as during and after the reaction. The dynamic mechanistic events, including primer terminus alignment, sugar pucker conformational changes, and transient metal ion binding, were found to play critical roles in DNA synthesis, repair, and fidelity control. Here, we reviewed recent static and dynamic structures of X- and Y-DNA polymerases with an emphasis on the power of time-resolved x-ray crystallography.

## DYNAMICS OF PRIMER TERMINUS ALIGNMENT

During DNA synthesis, the 3′-OH terminal group of the primer needs to align properly with respect to the Me^2+^ and the incoming nucleotide to undergo deprotonation and nucleophilic attack. Computational simulation studies have revealed that the primer has to be within 2.3–2.8 Å from the α-phosphate for efficient nucleophilic attack (Venkatramani and Radhakrishnan, 2010). A subtle movement or rotation in the primer by only 1–2 Å can place the primer 3′-O beyond this range and restrict chemistry. Moreover, the general base must approach the 3′-OH of the primer to carry out proton transfer before or concurrently during the nucleophilic attack. In order to capture ternary polymerase complexes, most early structural studies utilized DNA in which the primer strand contained dideoxynucleosides at the termini (Doublié *et al.*, 1998; Sawaya *et al.*, 1994). Without a functional 3′-OH group, these structures found that the Me^2+^_A_ was either missing or not properly aligned (Johnson and Beese, 2004; Vaisman *et al.*, 2005). Thorough examination of primer 3′-OH-Me^2+^_A_ alignment was first executed with nonhydrolyzable dNTP analogs with X family Pol β (Batra *et al.*, 2008) and Pol λ (Garcia-Diaz *et al.*, 2007), revealing that the primer 3′-O coordinates the Me^2+^_A_ as one of the ligands in the octahedral shell and exists 3.4 Å from the incoming nucleotide prepared for nucleophilic attack. However, exactly how and what docks the primer 3′-O into alignment and promotes its deprotonation and nucleophilic attack remained unclear.

**FIG. 2.**
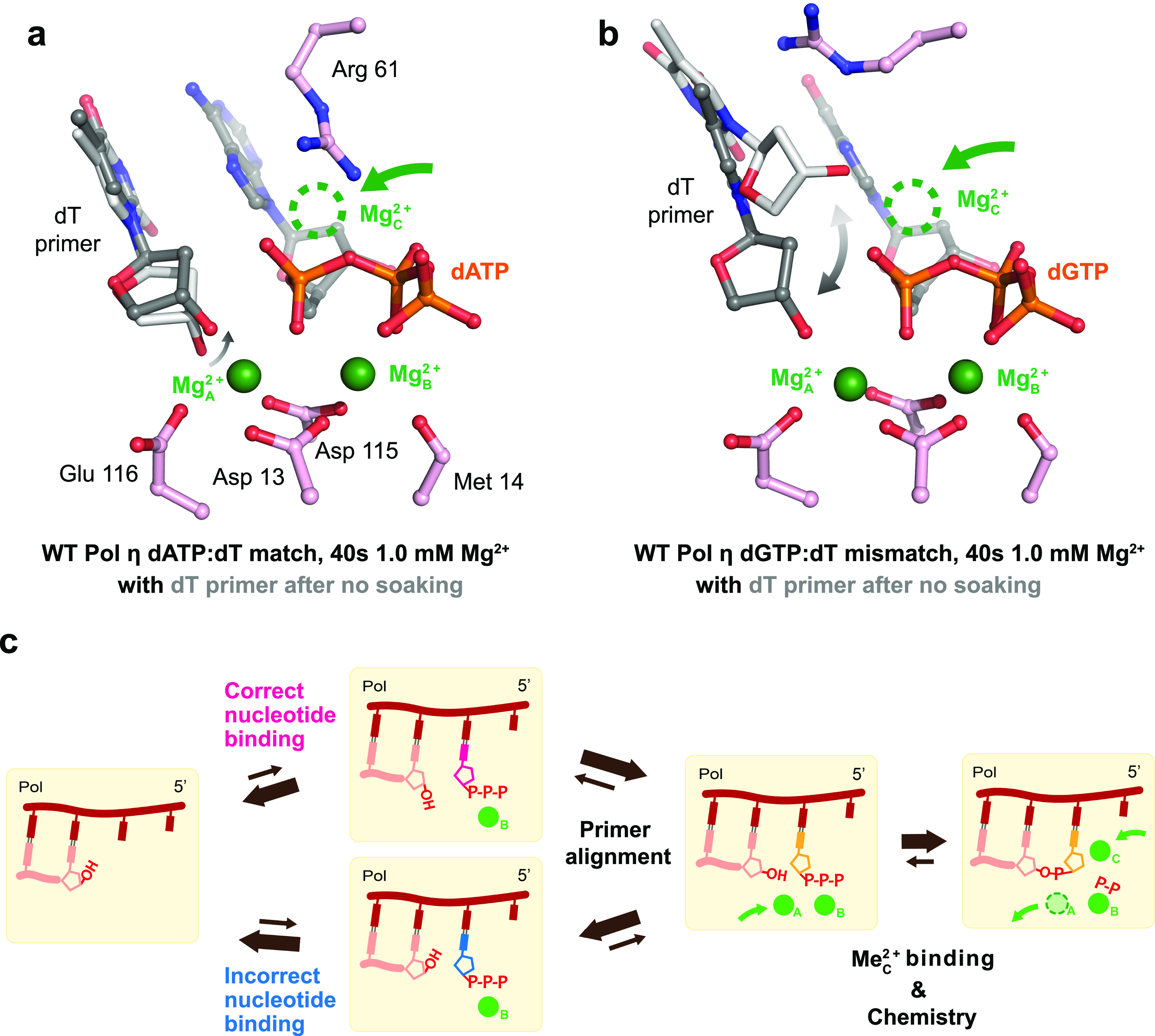
Primer terminus alignment during DNA synthesis. (a) Primer terminus alignment during correct incoming nucleotide incorporation prior to nucleophilic attack. Superimposition of the ternary structure of Pol η after 40 s 1.0 mM Mg^2+^ soaking (**4ECR**) in gray and the primer terminus with no soaking (**4ECQ**) in light gray during dATP incorporation across a dT indicates movement in the primer prior to nucleophilic attack. **b,** Primer terminus alignment during incorrect incoming nucleotide incorporation prior to nucleophilic attack. Superimposition of the ternary structure of Pol η after 40 s 1.0 mM Mg^2+^ soaking (**7U77**) in gray and the primer terminus with no soaking (**7U72**) in light gray during dGTP misincorporation across a dT indicates that the primer is dynamic prior to nucleophilic attack. The Mg^2+^_A_ and Mg^2+^_B_ are represented with solid green spheres, while the Mg^2+^_C_ binding site is represented with a dotted unfilled circle. (c) Proposed kinetic model during DNA polymerase correct nucleotide incorporation (red pink) and misincorporation (blue). During correct nucleotide incorporation, the primer 3′-OH becomes well-aligned by the Me^2+^_A_, and the reaction proceeds right after Me^2+^_C_ binding. Catalysis occurs in conjunction to Me^2+^_C_ binding and Me^2+^_A_ dissociation.

Recent time-resolved x-ray crystallographic studies with X-family Pol β (Freudenthal *et al.*, 2013; Kumar *et al.*, 2022; Reed and Suo, 2017; Vyas *et al.*, 2015), Pol λ (Jamsen *et al.*, 2022), and μ (Jamsen *et al.*, 2017), and Y-family Pol η (Nakamura *et al.*, 2012) uncovered transient events that occur during correct nucleotide incorporation. By complexing Pol η with the correct, natural nucleotide dATP and Ca^2+^, Nakamura and co-workers showed that in the ground state without the Me^2+^_A_, the primer lies 4.1 Å away from the α-phosphate group with a bond angle of 169°, slightly misaligned for nucleophilic attack [Fig. 2(a)]. Correlated with the binding of Mg^2+^ at the A-site, the 3′-OH of the primer was docked at 174° by the Mg^2+^_A_ in the C2′-endo conformation to lie 3.3 Å from the α-phosphate. Coupled with product formation, the deoxyribose sugar ring at the primer end changes from a C2′-endo to C3′-endo sugar pucker conformation (Nakamura *et al.*, 2012). Similarly, in X-family Pol β and Pol λ, the primer 3′-O shifted over 1 Å closer to the α-phosphate upon Ca^2+^/Na^1+^ to Mg^2+^/Mn^2+^ exchange at the Me^2+^_A_ binding site (Freudenthal *et al.*, 2013; Jamsen *et al.*, 2022; Kumar *et al.*, 2022). These results highlight the contribution of Me^2+^_A_ binding and its role on primer 3′-O alignment.

## DEPROTONATION OF THE 3′-OH TERMINAL GROUP OF THE PRIMER

What exactly promotes primer deprotonation and whether it concertedly occurs with nucleophilic attack remain unclear and critical in understanding the catalytic mechanism of polymerases. During the *in crystallo* reaction with X-family Pol λ, a water molecule lying close to the primer terminus was captured, suggesting its potential role to aid primer deprotonation (Jamsen *et al.*, 2022). In contrast, *in crystallo* reaction by Pol β did not capture a water molecule interacting with the 3′-OH of the primer (Freudenthal *et al.*, 2013). Instead, it was proposed that the amino acid side chain Asp256, which lies close to the primer, might be responsible for primer deprotonation. Computational studies indicate that Asp256 might facilitate primer deprotonation (Batra *et al.*, 2013). However, it is important to note that this amino acid residue also coordinates the Me^2+^_A_, and mutating Asp256 will disrupt Me^2+^_A_ binding and also indirectly affect deprotonation. In the study with Y-family Pol η, occurring simultaneously with Me^2+^_A_ binding, a water molecule approached from behind and around the 2′ carbon of the primer to possibly facilitate 3′-OH deprotonation (Nakamura *et al.*, 2012). The protein residue Ser113, which lies close to the primer but does not coordinate the Me^2+^_A_, has also been suggested to promote primer deprotonation. In such close proximity to the primer terminus, the water molecule or Ser113 can easily accept the primer 3′-O proton. However, simultaneously displacing the water molecule and mutating amino acid residue 113 from serine to an alanine did not reduce catalytic efficiency (Gregory *et al.*, 2021). Therefore, primer deprotonation in Pol η was proposed to most likely occur through multiple pathways, and a well-defined general base is not needed.

## PRIMER TERMINUS ALIGNMENT DURING INCORRECT SUBSTRATE DISCRIMINATION

During the incorporation of the incorrect nucleotide, the role of the Me^2+^_A_ and the importance of primer terminus alignment were further pronounced. In static crystal structures of Pol η with the incorrect nucleotide at the insertion site, the primer is positioned misaligned in the up-shifted conformation, restricted from attacking the α-phosphate (Zhao *et al.*, 2013). A water molecule coordinates the Me^2+^_A_ in place of the primer 3′-O. Notably, the degree of primer misalignment varies depending on the DNA sequence near the primer terminus, which corresponds to the different misincorporation efficiencies reflected in the kinetic assays (Zhao *et al.*, 2013). During the *in crystallo* reaction with Pol η, the majority of the primer terminus exists in the misaligned up-shifted conformation near the roof of the active site in the absence of Me^2+^_A_ (Chang *et al.*, 2022). Upon Mg^2+^ binding to the Me^2+^_A_ binding site, the primer terminus is docked in the aligned conformation [Fig. 2(b)]. Nevertheless, the primer terminus exists 2.5 Å away from the Me^2+^_A_, still 0.5 Å longer than the optimal distance for Mg^2+^ coordination, suggesting major barriers in primer deprotonation and alignment. Similarly, during the *in crystallo* reaction with Pol β incorporating a dATP across a dG template, the primer terminus showed weak electron densities (σ less than 3.0 r.m.s.d for the F_o_-F_c_ omit map), suggesting it is partially destabilized (Freudenthal *et al.*, 2013). The suboptimal coordination distance of the Me^2+^_A_ to the primer end and this misaligned conformation in both polymerase systems, which places the primer terminus 3′-O in an unfavorable position for nucleophilic attack, explain the significant decrease in catalytic activity during misincorporation. Furthermore, it was previously established that Mn^2+^ can promote error-prone synthesis of polymerases. Titrating the polymerases *in crystallo* revealed that Mn^2+^ was more optimal than Mg^2+^ at aligning the primer end in Pol η, and binding of Mn^2+^ linearly correlates with primer alignment even during incorrect nucleotide incorporation (Chang *et al.*, 2022). Such mutagenic property of Mn^2+^ was also previously hinted by static crystal structures of Pol λ. The structures of Pol λ incorporating dCTP against a dG revealed that Mn^2+^ promotes the sugar pucker to exist in the C3′-endo conformation as opposed to the C2′-endo conformation, aiding the primer 3′-O to exist even closer to the target α-phosphate (Garcia-Diaz *et al.*, 2007). These results further highlight a correlation between the Me^2+^_A_, primer alignment, and polymerase fidelity.

Rearrangements at the primer terminus and the Me^2+^_A_ coordination environment in the polymerase active site have been observed during DNA damage bypass and nucleotide analog drug insertion. Phenanthriplatin is a potential cisplatin-analog cancer drug that covalently links to DNA and obstructs DNA synthesis and transcription (Johnstone *et al.*, 2014; Kellinger *et al.*, 2013; Park *et al.*, 2012). Similarly, cyclopurines like 8,5′-cyclo-2′-deoxyadenosine also stall the DNA replication machinery (Kuraoka *et al.*, 2001). When Pol η contacts 8,5′-cyclo-2′-deoxyadenosine or phenanthriplatin on the DNA template, the primer was found to misalign, preventing nucleotide incorporation (Gregory *et al.*, 2014; Weng *et al.*, 2018). The primer end aligned with the incoming nucleotide, and bypass occurred only in the presence of mutagenic Mn^2+^. Similarly, the drug (-)-β-L-2′,3′-dideoxy-3′-thiacytidine (lamivudine) binding at the Pol β insertion site resulted in primer terminus misalignment and perturbation of the Me^2+^_A_ octahedral coordination geometry. These structural results were reflected in lamivudine's tighter binding affinity and 323-fold less efficient incorporation rate than the natural substrate dCTP (Vyas *et al.*, 2017). In Pol β, Arg283 stabilizes the syn-conformation of 8-oxo-2′-deoxyguanosine (8-oxodG) through hydrogen bonding on the template. Such interaction shifts 8-oxodG into a geometry that favors base-pair binding of dATP, allowing dCTP to be incorporated over dATP by only 2-folds. When Arg283 is mutated to Lys in Pol β, 8-oxodG existed in the anti-conformation and shifted the template upstream by 2 Å, tugging the primer away from the Me^2+^_A_ to exist 139° out of line with the α-phosphate unable to attack the incorrect nucleotide dATP (Freudenthal *et al.*, 2012). Thus, primer alignment might be an innate mechanism that safeguards against the bypass or incorporation of some damaged substrates.

Furthermore, chemical modifications on the sugar moiety of nucleotides can affect the dynamics of primer alignment and incorporation efficiency. When a ribonucleotide (rN), which has one additional oxygen atom on C2′ compared to a deoxynucleotide (dN), existed at the primer terminus, the primer terminus was found to be in the aligned conformation even in the absence of the Me^2+^_A_ during correct nucleotide incorporation of Pol η (Gregory *et al.*, 2021). Recent studies show that misincorporation is favored during Pol η-mediated extension from a rN (Chang *et al.*, 2023). Crystal structures with rN at the 3′-end of the DNA duplex showed improvement in primer alignment even when the incorrect dGTP nucleotide was bound at the insertion site across from a template dT. Not only was the primer terminus more favored in the docked position for nucleophilic attack, but also the sugar motifs of these rN existed in the C3′-endo sugar pucker conformation, which is the favored conformation in the product state, as the 3′-O of the primer lies closer to the target α-phosphate (Batra *et al.*, 2006; Berman *et al.*, 2007; Doublié *et al.*, 1998; Franklin *et al.*, 2001; Garcia-Diaz *et al.*, 2007; Li *et al.*, 1998; Nakamura *et al.*, 2012; Wang and Yang, 2009). Cytarabine (araC) is a dC mimic that contains a -OH group in the β direction on the 2′ carbon of the arabinose sugar moiety. Static structures show that at the primer terminus, araC exists in both aligned and misaligned conformations even during correct nucleotide incorporation. Moreover, this dC mimic drug exists in the C2′-endo conformation and misaligned with the α-phosphate, explaining how araC inhibits DNA extension even though it contains a functional 3′-OH group (Rechkoblit *et al.*, 2018). These results again showcase the importance of primer alignment and suggest small changes on the sugar ring can significantly impact incorporation efficiency.

## TRANSIENT METAL ION BINDING

Based on static crystal structures, Steitz proposed that two-metal ions are required and sufficient for polymerase catalysis (Steitz and Steitz, 1993). The Me^2+^_B_ stabilizes the incoming nucleotide by coordinating with its phosphate oxygens; the Me^2+^_A_ helps to decrease the pKa of the primer for deprotonation and nucleophilic attack. Recent *in crystallo* reaction studies revealed an additional divalent metal ion, or the C site metal ion (Me^2+^_C_) binds between the Pα and Pβ of the incoming nucleotide during reaction (Freudenthal *et al.*, 2013; Nakamura *et al.*, 2012; Vyas *et al.*, 2015; Yang *et al.*, 2016). Instead of protein sidechains, it is coordinated by four water molecules and two oxygens on the newly formed DNA strand and pyrophosphate. It has only been captured during catalysis but not detected in static structures with various inhibitors. Thus, the Me^2+^_C_ was not detected in decades of static structures of polymerases and remains hard to probe. The exact timing of Me^2+^_C_ binding and its role in DNA synthesis remain controversial. It has been proposed that the Me^2+^_C_ stabilizes the product state, pushes the reaction forward, inhibits the forward reaction, induces the reverse reaction, and/or helps with damage bypass.

**FIG. 3.**
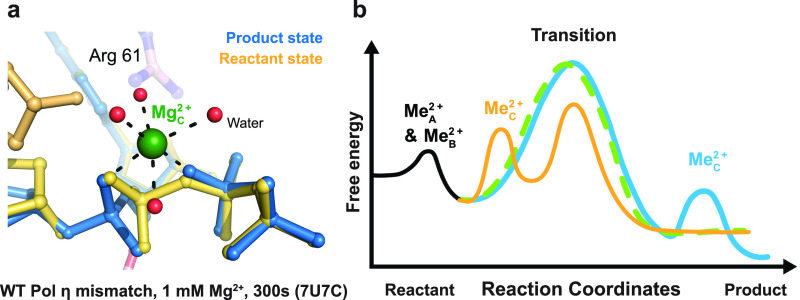
Geometry of the transient Me^2+^_C_ and its role during DNA synthesis. (a) The coordination of Mg^2+^_C_ in Pol η. The reactant state is colored in yellow, while the product state is colored in blue. The red spheres represent the water molecules that coordinate the octahedral shell of the Mg^2+^_C_ that is colored in green. (b) Proposed free energy diagram for the two-metal ion dependent (green) and three-metal ion dependent (yellow or blue) DNA synthesis. The Mg^2+^_C_ may bind prior to catalysis and drive the forward reaction (yellow) or bind after catalysis to stabilize the converted product (blue).

Initially, the Me^2+^_C_ has been suggested to bind to the newly formed DNA and pyrophosphate to stabilize the product state. In Pol η, the Me^2+^_C_ was captured simultaneously with production formation, except at the very beginning of the reaction (Nakamura *et al.*, 2012). In Pol β (Freudenthal *et al.*, 2013) and μ (Jamsen *et al.*, 2017), during correct nucleotide incorporation, the electron density of the Me^2+^_C_ appears slightly right after the detection of new bond density. Concurrently or immediately after, the Me^2+^_A_ dissociates from the active site. Thus, the role of the Me^2+^_C_ has been suggested to help reduce the electronegative buildup of the newly formed DNA backbone and also to help induce pyrophosphate protonation and release [Fig. 3(b)].

Moreover, the Me^2+^_C_ potentially drives the reverse reaction of phosphorolysis. The Me^2+^_C_ coordinates the newly formed DNA and pyrophosphate oxygens at 2.0–2.2 Å. Just like how the Me^2+^_A_ closely coordinates the primer terminus and helps to deprotonate the primer 3′-OH, the Me^2+^_C_ is optimally positioned to deprotonate one of pyrophosphate's oxygen. Once deprotonated, the more electronegative charged oxygen can undergo nucleophilic attack on the newly formed product phosphate to break the newly formed bond. Hence, it has been suggested that the role of the Me^2+^_C_ lies in deprotonating pyrophosphate (Freudenthal *et al.*, 2013). Nevertheless, phosphorolysis has never been visualized with time-resolved crystallography before, as the forward reaction is far more efficient than the reverse reaction. However, quantum mechanical/molecular mechanical simulations show that the Me^2+^_C_ can reduce the energy barrier for phosphorolysis to occur (Perera *et al.*, 2015).

Recent evidence suggests that the Me^2+^_C_ may be required to drive the DNA synthesis reaction. With Mn^2+^, which is more electron dense and, thus, can be detected at low occupancies, it was shown that Me^2+^_C_ binding perfectly correlates with production formation even during early stages of the reaction in Pol η (Gao and Yang, 2016). Recent studies with cross-linked Pol β also showed the Me^2+^_C_ binding simultaneously with phosphodiester formation (Kumar *et al.*, 2022). Moreover, extensive *in crystallo* titration with Pol η found that the A and B sites are high affinity binding sites (<0.5 mM), while the C site is a weak binding site with a low binding affinity of ∼3 mM, similar to what has been found in solution. When only the A site and B sites are saturated, the primer 3′-O, α-phosphate, and bridging oxygen co-linearize and are inclined for nucleophilic attack, but the reaction product is not observed even after long reaction time. Only after Me^2+^_C_ binding, product formation did occur, suggesting the essential role of the Me^2+^_C_ during catalysis. Furthermore, when a sulfur element, which is less electronegative than oxygen, replaces the incoming nucleotide's Sp oxygen, which serves as a coordinating ligand to the Me^2+^_C_, density surrounding the usual Me^2+^_C_ is not captured, and the Me^2+^ binding affinity is reduced by 25-fold from the kinetic assays (Gao and Yang, 2016). During slow mismatch incorporation, the transient Me^2+^_C_ was captured binding before product formation (Chang *et al.*, 2022). Initially, it binds between the Pα and Pβ oxygens but exists slightly too far from the Pα oxygen of the incoming nucleotide. After the Me^2+^_C_ enters the active site in between the Pα and Pβ oxygens and shifts downward to form an optimal octahedral geometry, the α-β-phosphate bond breaks. Thus, the *in crystallo* studies with Pol η suggested that Me^2+^_C_ binding proceeds catalysis, and that the Me^2+^_C_ may be directly involved in reducing the energy barrier for α-β-phosphate bond breakage [Fig. 3(b)].

This proposed catalytic role of the Me^2+^_C_ has been challenged in recent years. Wang and colleagues point out that because the occupancy of the Me^2+^_C_ is always lower than that of the product pyrophosphate, the Me^2+^_C_ must bind after reaction in the product complex and, thus, cannot actively push the catalytic reaction forward (Wang and Smithline, 2019). However, it has been argued that time-resolved experiments performed by hand cannot differentiate whether the Me^2+^_C_ binds before or after reaction, as Me^2+^_C_ binding can occur on the order of sub-seconds (Tsai, 2019). They also argued that the loss of the Me^2+^_C_ binding after product DNA and pyrophosphate formation could explain why the occupancy of the Me^2+^_C_ is always lower than that of the product pyrophosphate. While computational studies argue that Mg^2+^ or Mn^2+^ cannot favorably bind between the Pα and Pβ oxygens of the incoming nucleotide due to suboptimal bond lengths, geometry angle, and electrostatic charges of the oxygens, Chang and colleagues captured Mg^2+^ and Mn^2+^ binding near the Me^2+^_C_ binding site prior to reaction (Chang *et al.*, 2022). Furthermore, high concentrations of Mg^2+^ or Mn^2+^ used in *in crystallo* soaking experiments have been shown to inhibit the polymerase reaction in solution (Frank and Woodgate, 2007; Wang and Konigsberg, 2022). Thus, the Me^2+^_C_ has been suggested to remain bound with the product pyrophosphate within the active site. At this location, it can induce the reverse reaction and hold the product pyrophosphate within the active site. With the substrate unable to enter, additional DNA synthesis cannot occur at high metal ion concentrations.

It has also been proposed that transient divalent metals can help during the incorporation of damaged substrates. During the incorporation of the anti-form of 8-oxo-7,8-dihydro-2′-deoxyguanosine triphosphate (8-oxoGTP), which is sterically destabilized within the nucleotide binding pocket in Pol β, minimal distortion of the active site was observed. Instead, a divalent metal ion was captured binding near the Pα and Pβ oxygens prior to reaction to possibly alter the electrostatic nature of the α-phosphate oxygens to favor nucleophilic attack (Freudenthal *et al.*, 2015). However, another study on dCTP incorporation across an (anti) 8-oxoGTP found the Me^2+^_C_ only binds during the catalytic reaction (Vyas *et al.*, 2015). Similarly in X-family Pol μ, after crystals were soaked in 50 mM Mn^2+^, density close to the Me^2+^_C_ was captured and found coordinating the C8′ oxygen of 8-oxodGTP and a nearby aspartate indirectly through a water molecule (Jamsen *et al.*, 2021). Furthermore, an additional divalent metal site was captured near the Me^2+^_A_ binding site in Pol η during the incorporation of Sp-dATPαS, which has the Sp oxygen replaced with a sulfur element (Gao and Yang, 2016). While it remains unclear if these densities found in Pol β, μ, and η truly represent the Me^2+^_C_, it is likely that one additional or more transiently bound divalent metal ions play the role of stabilizing strained substrates for more efficient incorporation.

In addition to DNA polymerases, it has also been proposed that all RNA polymerases (Steitz, 1998), many nucleases (Stahley and Strobel, 2005; Toor *et al.*, 2009; Toor *et al.*, 2008), and ribozymes (Steitz and Steitz, 1993) follow the two-metal-ion dependent catalysis mechanism. Similar time-resolved crystallography experiments revealed an additional divalent Me^2+^ binding transiently during RNA cleavage in RNaseH (Samara and Yang, 2018). Moreover, the authors further discovered two additional monovalent Me^1+^ binding near the RNA backbone, possibly facilitating the water-mediated deprotonation and nucleophilic attack as well as stabilizing the transition state. The existence of the third Me^2+^ has also been suggested by studies on Endonuclease V, which was proposed to use a two-metal-ion dependent mechanism for cutting RNA. Similar as in RNaseH, a third divalent Me^2+^ binds transiently to direct the nucleophilic water molecule during Endonuclease V catalysis (Wu *et al.*, 2019). Furthermore, a transient Me^2+^ has also been sighted in MutT, which initially was proposed to catalyze 8-oxodGTP hydrolysis with two divalent Me^2+^ (Nakamura and Yamagata, 2022). The intermediate structures showed an additional metal ion binding in between two protein residues and directing a nucleophilic water closer and aligned to the scissile phosphate. In each of these three systems, the transient divalent Me^2+^ binds on the side of the nucleophilic water molecule, aligning it for nucleophilic attack. It is possible that many more metalloenzymes use transient metal ions during catalysis to stabilize substrate, product, and/or promote transition state formation.

## CONCLUSION

Time-resolved crystallography has extensively increased the understanding of DNA polymerases by revealing transient events like primer alignment, role of the Me^2+^_A_, and the presence of transient metal ions that occur during nucleotidyl transfer and DNA repair. However, *in crystallo* visualization of DNA synthesis remains limited to only X- and Y-family polymerases, which are specialized in DNA repair and damage bypass, possibly because polymerases that function during DNA replication such as A- and B-family polymerases perform catalysis too rapidly to be observed *in crystallo*. Furthermore, polymerases that exhibit large global conformation changes, diffract to sub-atomic resolutions lower than 3 Å, or are intolerant of cofactor soaking would present challenges for *in crystallo* studies. In addition, this manual metal ion diffusion technique is limited by how fast the crystallographer can feasibly transfer ternary complex crystals between buffer solutions, which is typically limited to 10 s. Automated stop flow equipment coupled with x-ray free electron lasers may further improve the temporal resolution of transient observations and lead to discoveries of new protein dynamic events before or during catalysis (Olmos *et al.*, 2018). Nevertheless, the number of diverse enzymes being studied with time-resolved crystallography continues to increase, highlighting the essential roles of active site dynamics and transient element binding during enzyme catalysis.

## Data Availability

The data that support the findings of this study are available from the corresponding author upon reasonable request.
